# Comparing the immunomodulatory properties of equine BM-MSCs culture expanded in autologous platelet lysate, pooled platelet lysate, equine serum and fetal bovine serum supplemented culture media

**DOI:** 10.3389/fvets.2022.958724

**Published:** 2022-08-25

**Authors:** Kayla M. Even, Angela M. Gaesser, Sarah A. Ciamillo, Renata L. Linardi, Kyla F. Ortved

**Affiliations:** Department of Clinical Studies, New Bolton Center, School of Veterinary Medicine, University of Pennsylvania, Kennett Square, PA, United States

**Keywords:** platelet lysate, immunomodulation, osteoarthritis, xenogen, mesenchymal stem cells

## Abstract

Joint injury often leads to cartilage damage and posttraumatic inflammation, which drives continued extracellular matrix degradation culminating in osteoarthritis. Mesenchymal stem cells (MSCs) have been proposed as a biotherapeutic to modulate inflammation within the joint. However, concerns have been raised regarding the immunogenicity of MSCs cultured in traditional fetal bovine serum (FBS) containing media, and the potential of xenogenic antigens to activate the immune system causing rejection and destruction of the MSCs. Xenogen-free alternatives to FBS have been proposed to decrease MSC immunogenicity, including platelet lysate (PL) and equine serum. The objective of this study was to compare the immunomodulatory properties of BM-MSCs culture-expanded in media supplemented with autologous PL (APL), pooled PL (PPL), equine serum (ES) or FBS. We hypothesized that BM-MSCs culture expanded in media with xenogen-free supplements would exhibit superior immunomodulatory properties to those cultured in FBS containing media. Bone marrow-derived MSCs (BM-MSCs) were isolated from six horses and culture expanded in each media type. Blood was collected from each horse to isolate platelet lysate. The immunomodulatory function of the BM-MSCs was assessed *via* a T cell proliferation assay and through multiplex immunoassay quantification of cytokines, including IL-1β, IL-6, IL-8, IL-10, and TNFα, following preconditioning of BM-MSCs with IL-1β. The concentration of platelet-derived growth factor BB (PDGF-BB), IL-10, and transforming growth factor-β (TGF-β) in each media was measured *via* immunoassay. BM-MSCs cultured in ES resulted in significant suppression of T cell proliferation (*p* = 0.02). Cell culture supernatant from preconditioned BM-MSCs cultured in ES had significantly higher levels of IL-6. PDGF-BB was significantly higher in APL media compared to FBS media (*p* = 0.016), while IL-10 was significantly higher in PPL media than ES and FBS (*p* = 0.04). TGF-β was highest in APL media, with a significant difference in comparison to ES media (*p* = 0.03). In conclusion, expansion of equine BM-MSCs in ES may enhance their immunomodulatory abilities, while PL containing media may have some inherent therapeutic potential associated with higher concentrations of growth factors. Further studies are needed to elucidate which xenogen-free supplement optimizes BM-MSC performance.

## Introduction

Osteoarthritis (OA) remains the leading cause of lameness in the equine industry, affecting approximately 60% of the equine athlete population, and often leads to early retirement or even euthanasia (1). Posttraumatic OA (PTOA) occurs secondary to acute or chronic joint trauma and is likely the most common type of OA in the horse. Joint trauma can cause global inflammation of the articular environment with increased synthesis of catabolic cytokines and degradative enzymes that cause progressive degeneration of the articular cartilage (2, 3). Currently, there are no effective disease-modifying drugs that halt or reverse OA in the horse with treatment regimens being aimed at reducing the clinical signs associated with OA.

Recent research has investigated the immunomodulatory properties of bone marrow-derived mesenchymal stem cells (BM-MSCs) to treat posttraumatic inflammation that occurs after injury. MSCs are easily accessible and amenable to culture expansion yielding large numbers of cells for intra-articular injection. MSCs have been shown to modulate the intra-articular environment through anti-inflammatory and trophic paracrine signaling (4, 5). They are also able to modulate the inflammatory cascade by suppressing T cell proliferation and polarizing pro-inflammatory macrophages toward an anti-inflammatory phenotype (6, 7). Additionally, MSCs recruit endogenous progenitor cells through chemokine production (7). Several studies have reported the benefit of MSC-mediated immunomodulation and secretion of bioactive factors in preclinical models of myocardial infarction (8), meniscal injury (9), and stroke (10).

Traditional culture expansion of MSCs utilizes fetal bovine serum (FBS) supplemented culture media which provides growth factors and nutrients (11, 12). However, concerns have been raised about the potential immunogenicity of FBS supplemented cells, with xenogenic antigens leading to immune system activation and cell rejection (13). Studies have demonstrated that both healthy and immunosuppressed equine patients commonly have anti-FBS antibodies, likely due to regular vaccinations (14). Others have demonstrated that FBS proteins can be internalized by and/or bound to the extracellular matrix of the MSCs during culture (15, 16). Even with methods that aim to remove FBS proteins, a detectable level of FBS proteins remain (15, 17). Additionally, Joswig et al. (17) found that horses treated with MSCs cultured in FBS until the time of intra-articular injection had increased total nucleated cell counts and increased lameness compared to horses treated with MSCs that underwent FBS depletion 48 h prior to injection (17).

Such findings have prompted investigation into culture media that is tailored to the equine species to minimize adverse reactions including commercially available serum-free supplements designed for human MSCs, autologous or commercially available equine serum, and autologous or pooled equine platelet lysate. However, the effect of alternative supplements on MSC proliferation rates, immunophenotype, and immunomodulatory properties must be considered. Clark et al. (18) found that expanding equine MSCs in serum-free media (StemPro® by Gibco) altered the immunomodulatory properties of the MSCs by decreasing secretion of prostaglandin E_2_ (PGE_2_) and increasing secretion of interleukin-10 (IL-10), while Schubert et al. (19) found that culturing equine MSCs in different lots of serum-free media (StemMACS™ MSC Expansion Media Kit XF by Meltenyi Biotech) altered MSC morphology and immunophenotype (18, 19). To our knowledge, the use of commercially available equine serum throughout culture expansion has not been studied.

Recent studies have also investigated the use of autologous platelet lysate (PL) as an alternative to FBS (11, 12, 20, 21). Platelet lysate is generated from platelet-rich plasma (PRP) and contains high concentrations of growth factors, including transforming growth factor-β (TGF-β), platelet-derived growth factors (PDGF), and vascular endothelial growth factor (VEGF) due to degranulation of platelet alpha granules (22). Media supplementation with PL does not appear to compromise MSC proliferation, viability, or potency when compared to FBS (11, 12, 23, 24). Importantly, several recent studies have also shown that PL has significant anti-inflammatory properties *in vitro* (12, 20). Autologous PL can have considerable variability regarding concentration and presence of growth factors and cytokines depending on age, sex, and hydration status. Pooled PL from several individuals can be used to capitalize on the natural variability of growth factors and cytokines that exists amongst horses (11, 20). Additionally, it can be analyzed in advance to ensure batch-to-batch consistency, and can be used immediately or stored.

To our knowledge, no studies have been performed comparing the effects of xenogen-free supplements including APL, PPL and ES on the immunomodulatory properties of equine BM-MSCs in comparison to BM-MSCs cultured in FBS. Therefore, the objective of this study was to compare the immunomodulatory properties of BM-MSCs culture-expanded in media supplemented with APL, PPL, ES and FBS. We hypothesized that BM-MSCs culture expanded in media with xenogen-free supplements would exhibit superior immunomodulatory properties to those cultured in FBS containing media.

## Materials and methods

### Animals

**Figure 1:**
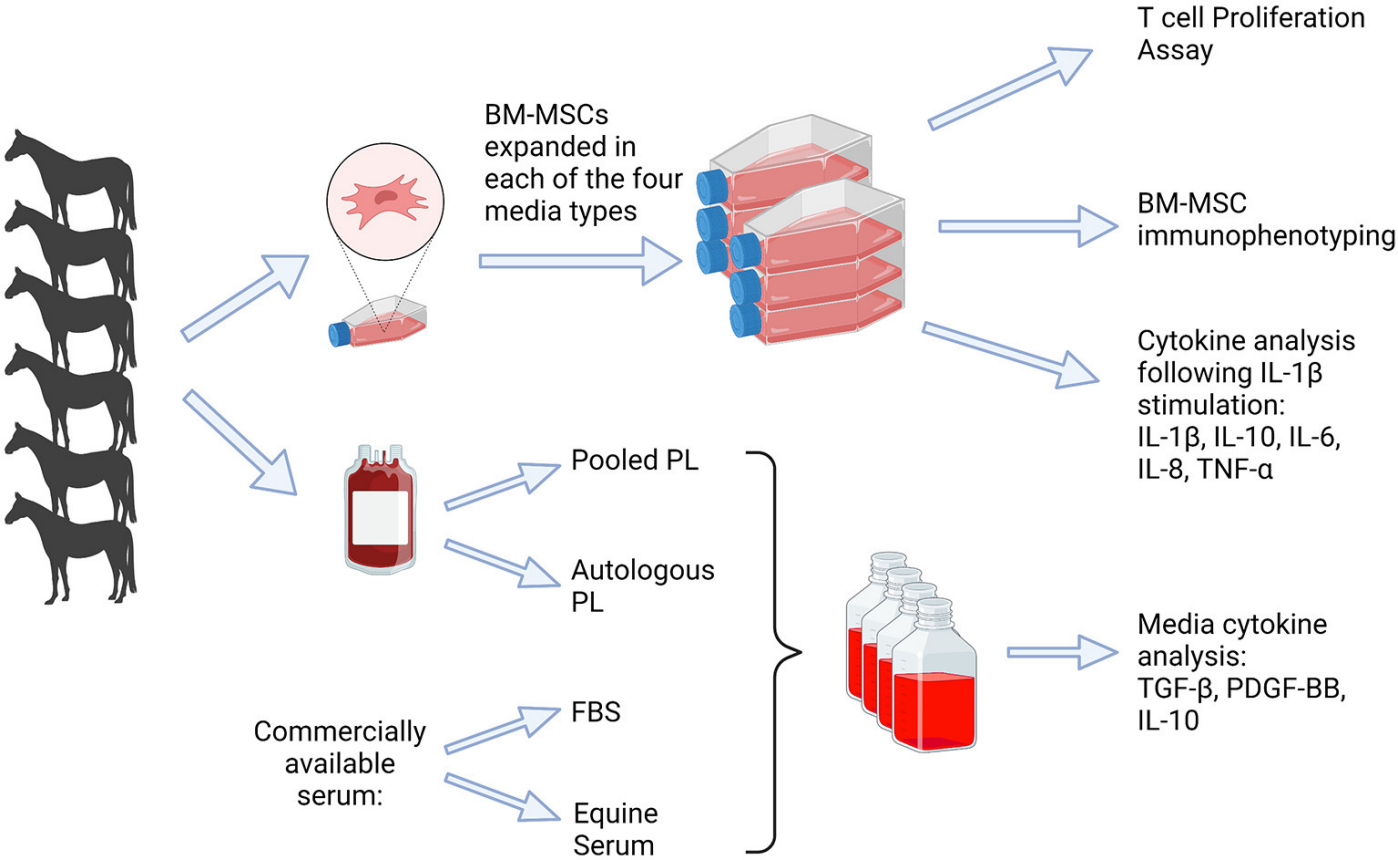
Depiction of study design. Blood was obtained (*n* = 6) to produce platelet lysate, which was used for APL and PPL containing media. Bone marrow was obtained for isolation of BM-MSCs and culture expanded in each of four media types, FBS, APL, PPL, and ES. Analyses included cytokine quantification in the media, T cell proliferation assay, BM-MSC immunophenotype, and cytokine analysis. FBS, fetal bovine serum; PL, platelet lysate; BM-MSCs, bone marrow-derived mesenchymal stem cells. This graphic was created with BioRender.com.

Six adult Thoroughbred horses (4–9 years) were used in this study. This group included 2 mares and 4 geldings weighing 475–525 kg. Prior to bone marrow aspiration, the horses were determined to be systemically healthy based on a thorough physical exam. The study was performed per Institutional and NIH guidelines for the Care and Use of Laboratory Animals and the study was approved by the Institutional Animal Care and Use Committee (IACUC) at the University of Pennsylvania. An overview of the study design is represented in Figure 1.

### Platelet lysate preparation

Platelet lysate was prepared as previously described (23). Briefly, venous blood was sterilely collected into blood collection bags containing anticoagulant citrate phosphate dextrose adenine (CPDA) for a total blood volume of 2,700 mL per horse. Blood was transferred to 50 mL conical tubes and centrifuged at 200 *g* with minimum acceleration and deceleration for 15 min. Plasma was aspirated from above the buffy coat, placed into new 50 mL conical tubes, and centrifuged at 400 *g* with minimum acceleration and deceleration for 15 min. Platelet-poor plasma (PPP) was then removed leaving ~1 mL of PRP. Platelet concentration was measured using an HT5 Element Hematology Analyzer (Heska Corporation, Loveland, CO). The PPP was then used to resuspend the platelet pellet to obtain a concentration of 1 x 10^12^ platelets/L. Platelet lysate was generated by a single freeze/thaw cycle at −80°C overnight followed by thawing at 37°C and centrifugation at 4,000 *g* for 15 min. To prepare PPL, an equal volume of PL from each horse (*n* = 6) was pooled together. All PL was stored at −20°C before use.

Mechanical fibrinogen depletion of APL and PPL was performed following the combination of APL or PPL with basic media. The combined media was incubated at room temperature for 4 h followed by overnight incubation at 4°C. The mixture was then incubated for 1 h at 37°C to allow complete fibrin clotting and then shaken vigorously to disrupt the clot. The media was centrifuged at 3,000 *g* for 10 min and filtered through a 0.22 μm syringe filter.

### Bone marrow collection and MSC culture

Bone marrow was collected aseptically from the sternebrae of horses using an 11-gauge Jamshidi bone marrow biopsy needle (VWR Scientific, Bridgeport, NJ). Horses were sedated using a combination of xylazine at 0.4 mg/kg IV once) (Rompun?, Bayer, Whippany, NJ) and detomidine at 0.01 mg/kg IV once (Dormosedan®, Zoetis, Parsippany-Troy Hill, NJ). The skin over the sternum was clipped and aseptically prepared and local anesthesia was achieved by injecting 2% mepivacaine subcutaneously at 0.4–0.8 mg/kg (Carbocaine-V, Zoetis, Parsippany-Troy Hill, NJ). A volume of 30 mL of bone marrow was aspirated into a 60 mL syringe containing 10,000 IU of heparin. Bone marrow samples were processed *via* density centrifugation with Ficoll-Paque Plus (GE Healthcare, Chicago, IL, USA) before seeding into flasks containing culture media consisting of Dulbecco's Modified Eagle Medium (DMEM) with 1 g/L of D-glucose, 2 mM L-glutamine, and 1 mM sodium pyruvate (ThermoFisher Scientific, Hampton, NH), penicillin (100 U/mL)-streptomycin (100 μg/mL) solution (Invitrogen, Carlsbad, CA), HEPES (ThermoFisher Scientific, Hampton, NH) and either: (1) 10% autologous PL (APL), (2) 10% pooled PL (PPL), (3) 10% FBS (VWR Life Science Seradigm, VWR, Radnor, PA), or (4) 10% equine serum (ES; New Zealand origin, ThermoFisher Scientific, Hampton, NH). Culture media contained human basic fibroblast growth factor (bFGF, 1 ng/mL) (Invitrogen, Carlsbad, CA), except for ES media which contained equine bFGF (1 ng/mL). Culture media supplemented with APL and PPL also contained heparin (2 IU/mL).

Media was changed every 48 h and cells were passaged when they reached ~80% confluency using Accutase® Cell Detachment Solution (Innovative Cell Technologies, Inc., San Diego, CA). Cell number and viability were determined using the Cellometer Auto 2,000 Cell Viability Counter (Nexcelom Bioscience, Lawrence, MA) and ViaStain™ AOPI staining solution (Nexcelom Bioscience LLC, Lawrence, MA).

### Media analysis

The concentration of TGF-β, IL-10, and PDGF-BB in each of the 4 medias were quantified using immunoassays. TGF-β1 was quantified using an anti-human fluorescent bead-based assay with cross-reactivity to equine TGF- β1 and IL-10 was quantified using a commercial equine-specific fluorescent bead-based assay (Luminex, Austin, TX). PDGF-BB was quantified using a commercial ELISA kit (R&D Systems, Minneapolis, MN) (20). Quantification of IL-10 followed the protocol stated in the subheading ‘Preconditiong of BM-MSCs'. A brief protocol for quantification of TGF-β1 is as follows. Samples used for TGF-β1 analysis were treated with 2μL of 1.0 N HCl for each 25μL of sample. Samples were then incubated at room temperature with shaking for 15 min. Samples were neutralized using 2μL of 1.0 N NaOH. From here, the quantification of TGF-β1 follows the previously stated protocol for a commerical equine-specific fluorescent bead-based assay (Luminex, Austin, TX). Parameters for TGF-β1 anaylsis on the MAGPIX® with xPONENT® software were set at 50 events per bead and a sample size of 50μL. A brief protocol for analysis of PDGF-BB using a commercial ELISA kit (R&D Systems, Minneapolis, MN) is as follows. To each well, 100μL of assay diluent was added, followed by 100μL of standard, control or sample to the appropriate well. The plate was sealed and incubated at room temperature for 2 h followed by 4 washes. Next, 200μL of conjugate was added to each well and the plate was incubated for 1.5 h at room temperature. The plate was washed 4 times. Then, 200μL of substrate solution was added to each well and incubated for 30 min covered in foil at room temperature followed by adding 50μL of stop solution to each well. The plate was then read at 450 nm with wavelength correction set to 540 nm.

### Characterization of BM-MSCs

The immunophenotype of the cell populations was evaluated using passage 3 (P3) BM-MSCs from all four medias by flow cytometry analysis using specific markers for stemness. Briefly, cells were collected using Accutase® Cell Detachment Solution (Innovative Cell Technologies, Inc., San Diego, CA), placed in 96-well-round-bottom plates (1 × 10^5^ cells/well), and washed twice with phosphate-buffered saline (PBS). Cell pellets were resuspended in PBS containing 10% normal goat serum and incubated at 4 °C for 20 min. Cells were then incubated with the primary antibodies at 4 °C for 45 min, rinsed twice with PBS, resuspended in the secondary antibody when appropriate, and incubated at 4 °C for 45 min. After the final rinse, the pellets were re-suspended in 200 μL of PBS before analysis. Cells were stained with anti-CD29, CD44, CD90, CD105, CD45, CD-79α, major histocompatibility complex I (MHC I), and major histocompatibility complex II (MHC II) antibodies and isotype controls were used to establish fluorescent gates based on previously reported data (Table 1) (25). A 13-color, 4-laser Flow Cytometer instrument was used and subsequent analyses were performed using CytExpert v2.4 (Beckman Coulter, Brea, CA).

**Table 1:** Antibodies used for flow cytometric analysis of equine cells surface markers.

**Antibody**	**Clone/isotype**	**Host species**	**Target species**	**Fluorochrome**	**2°Antibody**	**Company**	**Dilution for 1°antibody**
CD29	TMD29/IgG1^a^	Mouse	Human	APC	Yes (Goat Anti-Mouse IgG)	EMD Millipore	1:100
CD44	IM7/IgG2b^b^	Rat	Human	FITC	No	Thermo IM7	1:80
CD90	HR-DH24A/IgM	Mouse	Canine, Equine	RPE	No	WSU Monoclonal Antibody Center	1:200
CD105	SN6/IgG1^b^	Mouse	Human	Alexa 488	No	BioRad	1:10
CD45RB	HR-DH16A/IgM	Mouse	Equine	RPE	No	WSU Monoclonal Antibody Center	1:200
CD79α	HM57/ IgG1^c^	Mouse	Human	Alexa 647	No	BioRad	1:200
MHCI	cz3/IgG2b	Mouse	Equine	APC	Yes (Goat Anti-Mouse IgG)	Gift^d^	1:100
MHCII	cz11/IgG1	Mouse	Equine	APC	Yes (Goat Anti-Mouse IgG)	Gift^d^	1:200
**Isotype Control**	**Corresponding MAB**		**Target Species**	**Fluorophore**		**Company**	**Dilution**
IgG1	To CD29		Mouse	APC		Abcam	1:100
IgG2b	To CD44		Rat	Alexa 488		Abcam	1:100
IgM	To CD90		Mouse	PE		Abcam	1:101
IgG1	To CD105		Mouse	Alexa 488		Abcam	1:200
IgM	To CD45RB		Mouse	PE		Abcam	1:200
IgG1	To CD79α		Mouse	Alexa 647		Abcam	1:400
IgG2b	To MHCI		Mouse	APC		Abcam	1:100
IgG1	To MHCII		Mouse	APC		Abcam	1:100

a Validated by Laval et al. (26).

b Validated by Paebst et al. (27).

c Validated by De Schauwer et al. (28).

d Gifts from Dr. Doug Antczak, Cornell University, Ithaca, New York, USA.

### T cell proliferation assay

Blood was collected from the same 6 horses into syringes containing anticoagulant citrate dextrose (ACD) and peripheral blood mononuclear cells (PBMCs) were isolated *via* Ficoll-Paque Plus centrifugation. PBMCs were labeled with carboxy-fluorescein diacetate succinimidyl ester (CFSE) fluorescent labeling solution (ab113853, Abcam, Cambridge, MA) as per the manufacturer's protocol. Briefly, PBMCs (50 x 10^6^ per mL of PBS) were labeled with 5 mM of CFSE at a 1:1 ratio of CFSE and PBS solution in a 15 mL conical tube. The tube was kept in the dark at room temperature, rotating for 5 min. To stop the reaction, an equal volume of FBS was added to the CFSE/PBS solution and mixed in the dark at room temperature for 1 min. The tube was then centrifuged for 5 min at 309 g at room temperature. PBMCs were washed twice with T cell media. PBMCs (2.5 x 10^6^ cells/well) were then plated alone or with autologous P3 BM-MSCs at an MSC:T cell ratio of 1:50 in a 24-well-plate with T cell medium consisting of RPMI (ThermoFisher Scientific, Hampton, NH), penicillin (100 U/mL)-streptomycin (100 μg/mL) solution (Invitrogen, Carlsbad, CA), 0.1 mM 2-mercaptoethanol (Sigma Aldrich, St. Louis, MO) and either: (1) 10% APL, (2) 10% PPL, (3) 10% ES, or (4) 10% FBS. T cell medium containing APL or PPL also contained heparin (2 U/mL). Cells were stimulated with concanavalin A (Con-A, 5 μg/mL, Sigma Aldrich, St. Louis, MO) for 5 days. PBMCs were then collected and placed (1.2 × 10^4^) in 96-well-plates, washed twice with PBS, resuspended in 100 μL of 10% normal goat serum (Sigma Aldrich, St. Louis, MO), and incubated at 4 °C for 20 min. Cells were first incubated with 50 μL of the anti-equine CD3 primary antibody (1:20 dilution, Clone UC-F6G, University of California, Davis, CA) at 4 °C for 45 min, rinsed twice with PBS, and then incubated with a secondary allophycocyanin (APC) labeled goat anti-mouse IgG antibody (100 μL, 1:100 dilution, Cat#550826, BD Biosciences, Franklin Lakes, NJ) at 4 °C for 45 min. After the final PBS rinse, the pellets were re-suspended in 200 μL of PBS containing 7-AAD (7-Aminoactinomycin D, ThermoFisher scientific, Waltham, MA) as a viability stain. The proliferation of CD3+ T cells was measured by flow cytometry using CFSE quantification. Unstimulated, CFSE-labeled, fresh PBMCs were used as a control to determine the amount of baseline proliferation of PBMCs maintained in culture for 5 days. Labeled and unlabeled PBMCs were used to set the positive and negative gates for CFSE, respectively. Unstimulated PBMCs were also used to set the gate of non-proliferating cells and Con-A stimulated PBMCs served as an internal control for proliferation. CD3+ T cells were identified by APC fluorescence. The percentage of proliferation events was based on the percentage of all APC + events and calculated in comparison to the internal control for each experiment. The gating strategy used in the T cell proliferation assays was performed as previously described (29).

### Preconditioning of BM-MSCs

Passage 3 BM-MSCs (50,000 cells/well) were plated in 24-well-plates in their respective media. After 24 h, fresh media consisting of Dulbecco's Modified Eagle Medium (DMEM) with 1 g/L of D-glucose, 2 mM L-glutamine, and 1 mM sodium pyruvate (ThermoFisher Scientific, Hampton, NH), penicillin (100 U/mL)-streptomycin (100 μg/mL) solution (Invitrogen, Carlsbad, CA), and HEPES (ThermoFisher Scientific, Hampton, NH) with or without recombinant equine interleukin-1β (IL-1β; 10 ng/mL, R&D Systems, Minneapolis, MN) was added and cells were incubated for an additional 48 h. After 48 h, supernatants were collected and stored at −20°C for later analysis. The concentration of supernatant immunomodulatory cytokines including IL-10, interleukin-6 (IL-6), IL-1β, interleukin-8 (IL-8), and tumor necrosis factor- α (TNF-α) were quantified using a commercial equine-specific fluorescent bead-based multiplex assay (Luminex, Austin, TX). The fluorescent bead-based multiplex assay was performed according to the manufacturer's protocol using the MAGPIX® instrument (Luminex, Austin, Tx). A brief protocol for the quantification of IL-10, IL-6, IL-1β, IL-8, and TNF-α is as follows. For each standard, control, and sample, 25μL was added to the appropriate well. The standard curve was generated using media containing Dulbecco's Modified Eagle Medium (DMEM) with 1 g/L of D-glucose, 2 mM L-glutamine, and 1 mM sodium pyruvate, penicillin (100 U/mL)-streptomycin (100 μg/mL) solution, and HEPES. To each well, 25μL of antibody-immobilized beads were added. The plate was sealed, wrapped in foil and placed on a shaker to be incubated overnight (16–18 h) at 4°C. The following day, the plate was washed 3 times, followed by adding 25μL of detection antibody to each well. The plate was covered, wrapped in foil and incubated at room temperature on a shaker for 1 h. Next, 25μL of streptavidin-phycoerythrin was added to each well-containing detection antibody and incubated for 30 min on a plate shaker at room temperature. The plate was then washed 3 times. Then, 150μL of drive fluid was added. Parameters for analysis on the MAGPIX® with xPONENT® software were set at 50 events per bead and a sample size of 100μL.

### Statistical analysis

Normality was assessed by visual inspection of histograms for a Gaussian distribution and a Kolmogorov-Smirnov test prior to statistical analysis. Parametric quantitative data are presented as mean ± SEM. Non-parametric quantitative data are presented as median (range). Differences between growth factors in media were determined either using a one-way analysis of variance (ANOVA) followed by Tukey's multiple comparison test for parametric data or using a Kruskal-Wallis ANOVA followed by Tukey's multiple comparison test for non-parametric data. For analysis of T cell proliferation, the proliferation of stimulated PBMCs cultured alone and stimulated and unstimulated PBMCs co-cultured with MSCs was normalized by dividing proliferation of unstimulated PBMCs cultured alone in the respective media type (Relative T cell proliferation %). Comparisons were made within and across all groups and conditions. Differences in T cell proliferation and supernatant cytokine quantification were determined using a mixed effects model with media type and stimulation as fixed effects and horse as a random effect. Statistical analysis was performed with JMP 12 (SAS, Cary, NC) software and the level of significance was set at *p* ≤ 0.05.

## Results

### Media analysis

**Figure 2:**
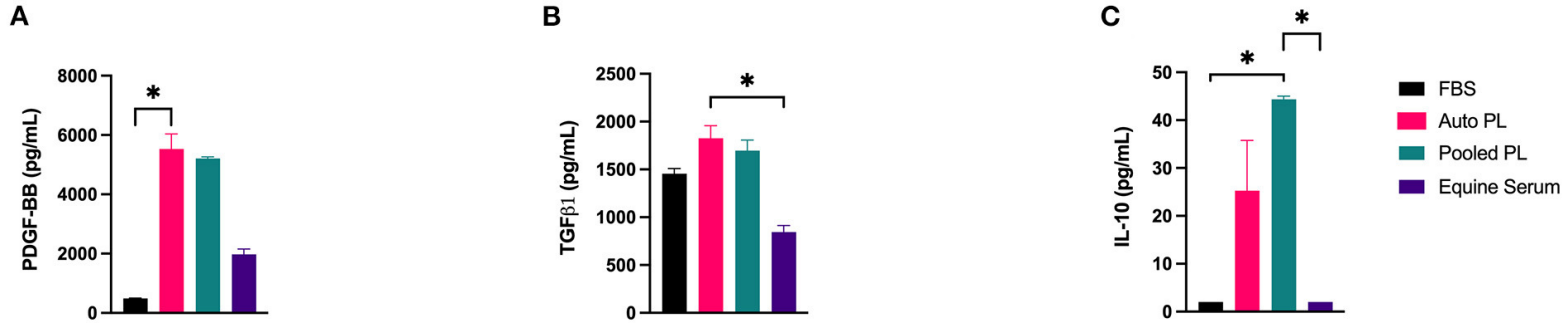
Mean (±SEM) concentration of **(A)** PDGF-BB, **(B)** TGF-β1, and **(C)** IL-10 in four different media types. Asterisks represent significant differences (*p* ≤ 0.05) (*n* = 6). Auto, autologous; PL, platelet lysate; FBS, fetal bovine serum; pg/mL, picograms per milliliter.

Using immunoassays, PDGF-BB, TGF-β1, and IL-10 were quantified in each of the four media types, the results of which are shown in Figure 2. PDGF-BB (Figure 2A) was highest in APL and PPL media. The concentration of PDGF-BB was significantly higher than in APL when compared to FBS media (*p* = 0.016). TGF-β1 (Figure 2B) concentration was highest in APL media and was significantly higher in APL media when compared to ES media (*p* = 0.033), which had the lowest concentration. IL-10 (Figure 2C) concentration was highest in PPL media, and this was significantly higher than FBS (*p* = 0.043) and ES (*p* = 0.043) media.

### Immunophenotype of BM-MSCs

**Figure 3:**
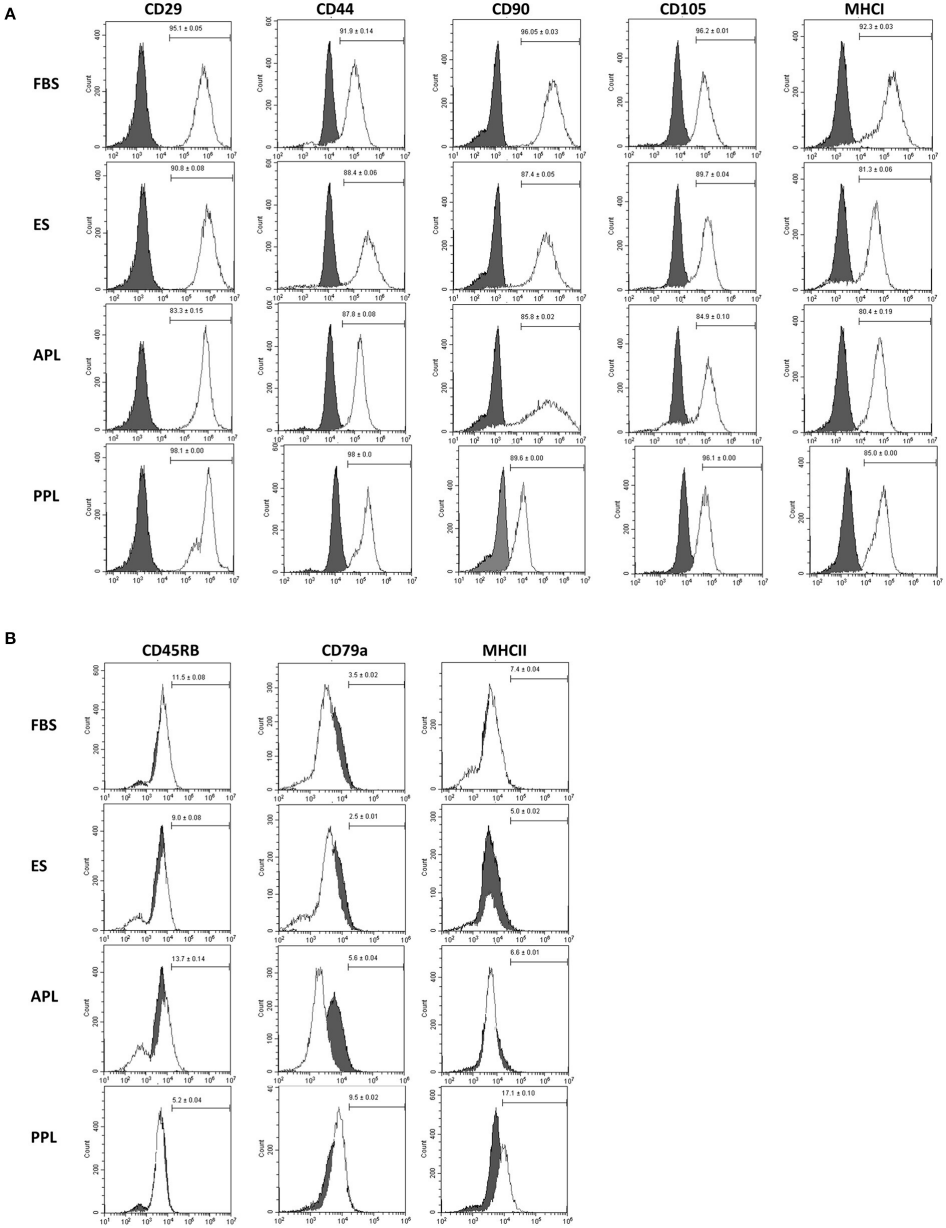
Characterization of P3 BM-MSCs cultured in FBS, APL, PPL, and ES supplemented media using flow cytometric quantification of cell surface marker expression. **(A)** Expression of inclusion markers in BM-MSC populations and **(B)** expression of exclusion markers in BM-MSC populations. The gray histograms represent isotype controls and white histograms represent respective cell surface marker staining. The mean (±SEM) percentage of positive cells, obtained from 6 horses with each horse providing 2 experimental replicates, is in the right corner of each histogram. The histogram is a representative result of these replicates. FBS, fetal bovine serum; APL, autologous platelet lysate; PPL, pooled platelet lysate; ES, equine serum.

Flow cytometric analysis of P3 equine BM-MSCs demonstrated similar immunophenotype among all cells supplemented with different media types (10% APL, 10% PPL, 10% ES, and 10% FBS). BM-MSCs cultured in all media types were strongly positive for markers of stemness including CD29, CD44, CD90, CD105 and MHCI (Figure 3A). Minimal expression of exclusion markers was present in all media types including CD45RB, CD79α, and MHCII (Figure 3B). No significant differences in expression of any cell surface marker were noted across all groups. Microscopically, all cells exhibited similar plastic adherence and fibroblast-like morphology.

### T cell proliferation assay

**Figure 4:**
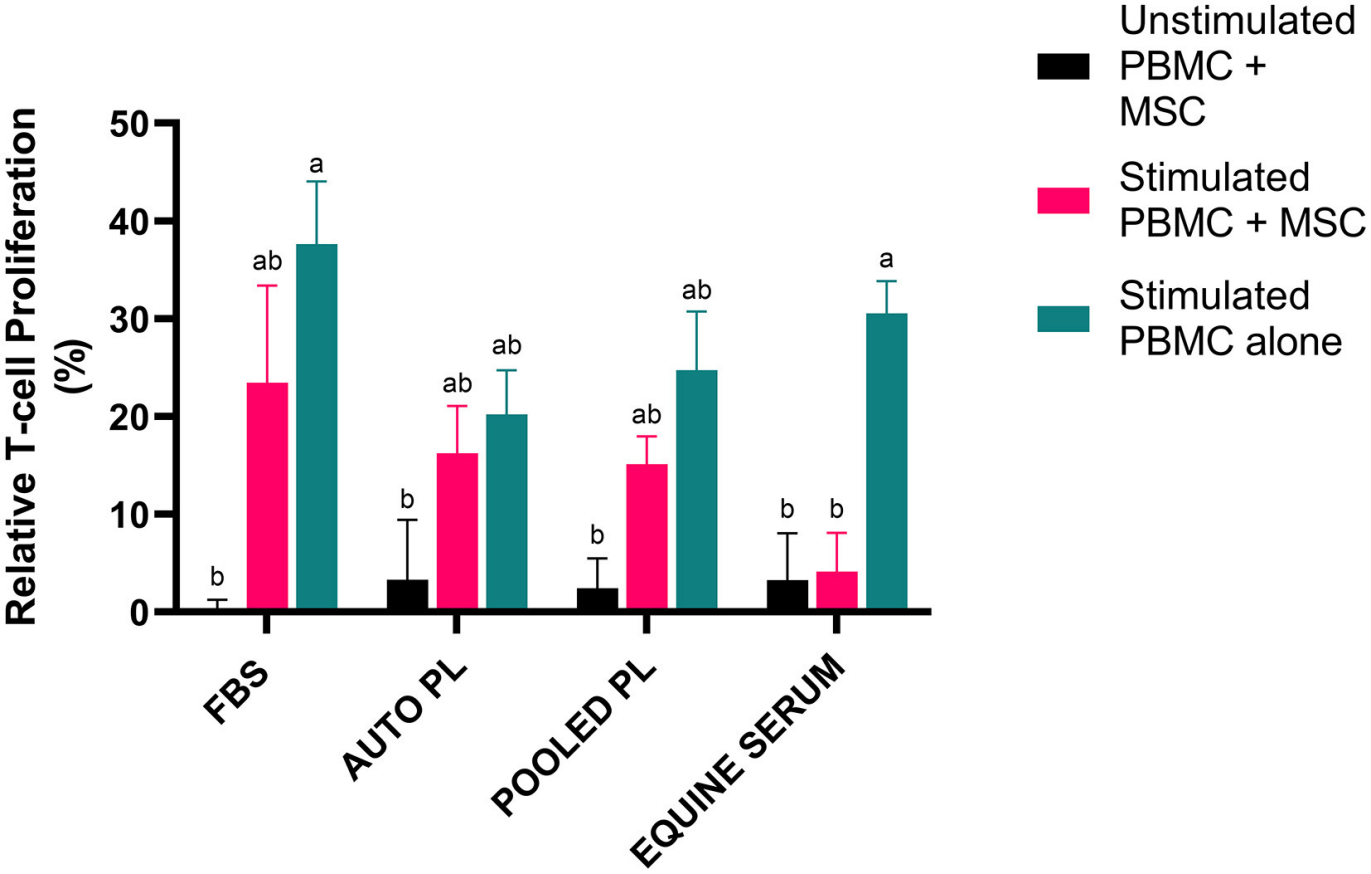
Representation of mean (±SEM) proliferation of CD3+ T cells as measured by flow cytometry using CFSE quantification. Peripheral blood mononuclear cells (PBMCs) were co-cultured with BM-MSCs in each media type and stimulated with (stimulated) or without (unstimulated) Con A. Proliferation was normalized by dividing by proliferation of unstimulated PBMCs cultured alone in the respective media type (Relative T cell proliferation %). Comparisons were made within and across all groups and conditions. Different letters denote significant differences between groups; *p* ≤ 0.05 (*n* = 6). FBS, fetal bovine serum; PL, platelet lysate; auto, autologous.

To assess the effect of the different media types on T cell proliferation, PBMCs were co-cultured with BM-MSCs and CFSE dilution was measured *via* flow cytometry to quantify PBMC proliferation. The results of the T cell proliferation assay are shown in Figure 4. Following stimulation, PBMCs cultured alone had the highest degree of proliferation in all media types. Co-culture with MSCs decreased proliferation in all groups. Suppression of T cell proliferation was most notable when PBMCs were co-cultured with BM-MSC cultured in ES media, compared to other media types, with T cell proliferation similar to unstimulated controls. Interestingly, APL and PPL media itself appeared to effect T cell proliferation as noted by decreased proliferation in stimulated PBMCs alone when compared to stimulated PBMCs alone cultured in FBS.

### Cytokine analysis

**Figure 5:**
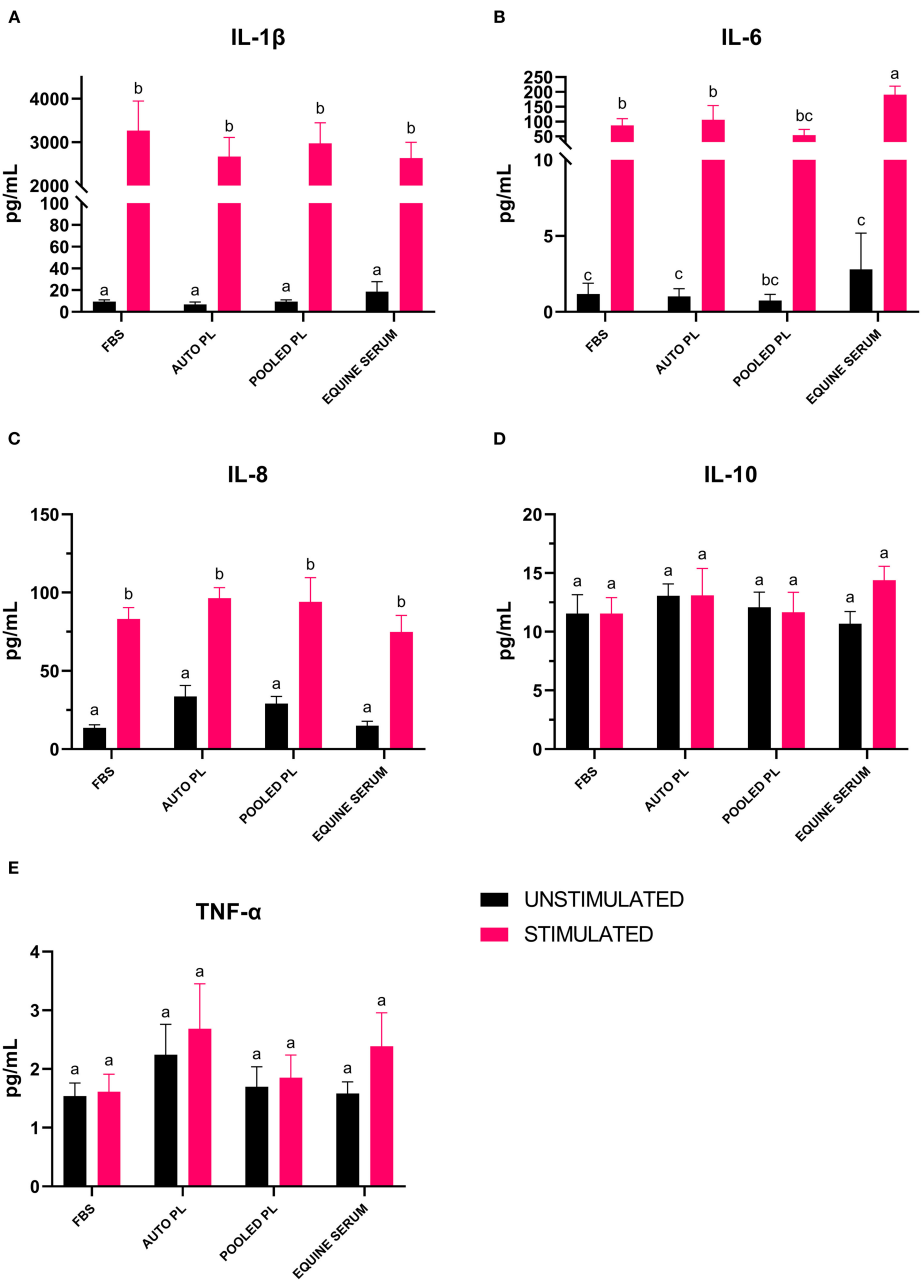
Mean (±SEM) concentration of **(A)** IL-1β, **(B)** IL-6, **(C)** IL-8, **(D)** IL-10 and **(E)** TNF-α in cell culture supernatants of BM-MSCs cultured in four different media types with (stimulated) or without (unstimulated) IL-1β preconditioning. Different letters denote significant differences between groups; *p* ≤ 0.05 (*n* = 6). PL, platelet lysate; EQ, equine; FBS, fetal bovine serum; auto, autologous; pg/mL, picograms per milliliter.

Following the culture of BM-MSCs in four different media types, cells were preconditioned with IL-1β. The supernatants were then analyzed using a fluorescent bead-based multiplex assay for quantification of IL-1β, IL-10, IL-6, IL-8, and TNF-α. For IL-1β (Figure 5A) and IL-8 (Figure 5C), there were significant increases in the concentration of the cytokines in supernatants from stimulated cultures, but there were no significant differences between media types. For IL-10 (Figure 5D) and TNF-α (Figure 5E), there were no significant differences between supernatants from stimulated and unstimulated cultures or between media types. There was a significant increase in IL-6 (Figure 5B) concentration in supernatants following preconditioning in all media types except PPL, however, IL-6 concentration was significantly higher in ES stimulated cultures when compared to APL (*p* = 0.034), PPL (*p* = 0.0001) and FBS (*p* = 0.0008).

## Discussion

The objective of this study was to compare the effects of autologous PL, pooled PL, and ES media supplementation on the immunomodulatory properties of BM-MSCs in comparison to traditional FBS media supplementation, with the hypothesis that that BM-MSCs culture expanded in media with xenogen-free supplements would exhibit superior immunomodulatory properties to those cultured in FBS containing media. BM-MSCs cultured in all media types had similar immunophenotypes as demonstrated by cell surface marker expression. Overall, we did not find any significant impact of PL, autologous or pooled, on the immunomodulatory properties of BM-MSCs, including suppression of T cell proliferation or synthesis of immunomodulatory cytokines. BM-MSCs cultured in ES media had significantly decreased T cell proliferation while having significantly more IL-6 produced following IL-1β preconditioning. Interestingly, APL and PPL media themselves modulated T cell proliferation to some degree when compared to FBS.

BM-MSCs cultured in all media types displayed the expected markers of stemness. Interestingly, cells cultured in PPL had a higher expression of MHCII than the other media types, although this difference was not significant. This finding corresponds to that of Yaneselli et al. (21), wherein the culture of equine BM-MSCs in allogeneic PL resulted in an increase in MHCI and MHCII gene expression in comparison to MSCs cultured in FBS (21). The role of MSC expression of MHCII has not been fully elucidated. MHCII expression has been associated with increased T cell proliferation suggesting increased immunogenicity (30). Increased expression of MHCII has also been implicated as a cause of humoral immune response to repeated intra-articular administration of allogeneic BM-MSCs (31). In contrast, Cassano et al. (32) found that MSCs primed with interferon gamma (IFN-γ), which exhibited upregulation of MHC II expression, had superior immunomodulatory abilities including suppression of T cell proliferation (32).

BM-MSCs cultured in ES supplemented media resulted in the highest degree of suppression of T cell proliferation when co-cultured with PBMCs such that T cell proliferation was similar to that observed in unstimulated control cultures. While suppression of T cell proliferation in APL and PPL supplemented BM-MSC co-cultures was similar to suppression noted in FBS supplemented co-cultures, we did find that T cell proliferation in APL and PPL supplemented cultures containing stimulated PBMCs alone was decreased compared to FBS supplemented cultures. This suggests the behavior of PBMCs can be altered by factors within the media itself independent of the effect of BM-MSCs. Several studies have shown that PDGF plays a role in T cell regulation (33) which may explain in part why there was suppression of T cell proliferation in our study compared to FBS. Although we expected enhanced suppression of T cell proliferation by BM-MSCs cultured in APL and PPL supplemented media, previous studies have found somewhat conflicting results when examining the effect of PL on BM-MSCs. Yaneselli et al. (21) found that BM-MSCs cultured in PL media had increased expression of immunomodulatory genes, while Naskou et al. (11, 12) found that BM-MSCs cultured in pooled PL did not enhance monocyte suppression compared to FBS (12, 21).

A standardized protocol for PL preparation has not been established. It is possible that conflicting reports regarding the effect of PL are due to differences in PL generation parameters. Debate remains as to how centrifugation speed, number of centrifugations, and number of freeze-thaw cycles in different PL protocols affect cytokine release from platelets (34–36). The PL generation protocol used in our study was developed by Russell et al. (23) where it was concluded that a single freeze-thaw cycle produced the highest concentrations of PDGF-BB and TGF-β (23). It is also possible that the addition of heparin to PL supplemented cultures may affect BM-MSCs. One study found that a minimum of 0.61 IU/mL of heparin was required to prevent gel formation in the culture media, while concentrations higher than 0.61IU/mL decreased the proliferation rate of MSCs, but morphology and immunophenotype were not altered (37). A recent study from Laner-Plamberger et al. (38), also found that human BM-MSCs cultured in human PL supplemented with 2 IU/mL of heparin exhibited upregulated gene expression for the signaling pathways relating to WNT, PDGF, NOTCH and TGF-β (38).

*In vivo*, MSCs are activated locally by inflammatory mediators and participate in modulation of inflammation. *In vitro*, preconditioning with inflammatory cytokines such as IFN-γ, TNF-α, IL-1β, or a combination of cytokines has been shown to induce their immunomodulatory properties and may enhance their survival *in vivo* (39–42). However, there is not a clear consensus as to which inflammatory mediators produce a consistent response without compromising MSC viability. Our preliminary experiments (results not shown) demonstrated IL-1β produced a more consistent activation of BM-MSCs following preconditioning compared to IFN-γ and/or TNF-α. To that end, we found that preconditioning with IL-1β led to a significant increase in IL-1β and IL-8 across all media groups, and for IL-6, there was a significant increase in all groups except for PPL. One limitation of measuring IL-1β in the supernatant is it is possible some recombinant IL-1β remained after 48 h. Ferreira et al. (43) also demonstrated upregulation of IL-1β in the secretome following preconditioning with IL-1β using a human cytokine array. However, when bovine intervertebral discs (IVD), which were pretreated with IL-1β to induce an inflammatory environment, were exposed to the MSC secretome, IL-1β was not upregulated in the IVD conditioned media (43). This suggests that the increase in IL-1β in the MSC secretome was not due to the presence of recombinant IL-1β alone. IL-1β plays an important role in the pathogenesis of OA, driving the production of catabolic factors such as matrix metalloproteinases (MMP-1, MMP-3 and MMP-13), IL-6, IL-8, monocyte chemoattractant protein 1 (MCP-1) and CC motif chemokine ligand 5 (CCL5) (44). Thus, this increase in IL-1β production by MSCs following preconditioning may be undesirable.

The consistent increase in IL-8 we observed following preconditioning is also of note. Hacket et al. (45) demonstrated an increase of similar magnitude when MSCs were preconditioned with 10 ng/mL IL-1β. They also showed a stronger induction of IL-8 when IL-1β was added to IFN-γ and TNF-α. IL-8 is elevated in OA tissues and is also a potent chemoattractant which promotes polymorphonuclear neutrophil (PMN) recruitment, participates in the regulation of natural killer cells, and activates T cell migration (44, 46–49). Again, increased production of pro-inflammatory cytokines by MSCs may be undesirable, however the behavior of IL-1β preconditioned MSCs *in vivo* is yet to be elucidated.

When preconditioned with IL-1β, we discovered that the supernatant from MSCs cultured in ES supplemented media contained a significantly higher concentration of IL-6 when compared to APL, PPL and FBS supplemented media. While IL-6 is generally categorized as a pro-inflammatory cytokine in OA pathogenesis, it also has some anti-inflammatory and immunomodulatory functions. Dorronsoro et al. (50) found that when IL-6 MSC production was silenced, MSCs showed a reduced ability to suppress activated T-cell proliferation. They also demonstrated that treatment of MSCs with IL-6 and other inflammatory cytokines improved the production of TGF-β while preserving immunomodulatory function (50). Some have argued that in the presence of TGF-β, IL-6 plays a key role in determining whether regulatory T cells (Treg) or Th17 T-cells are produced (51). Production of IL-6 by MSCs is also an important mediator of macrophage polarization with IL-6 resulting in upregulation of the anti-inflammatory M2b phenotype (52). Considering BM-MSCs cultured in ES media were the most effective at suppressing T cell proliferation, the effect of IL-6 on the immunomodulatory capacity of BM-MSCs when cultured in ES should be further investigated.

Platelet lysate supplemented medias contained higher levels of PDGF-BB, TGF-β and IL-10 in comparison to ES and FBS supplemented media. In addition to suppressing T cell proliferation, PDGF also increases MSC migration and proliferation. Endo et al. (53) found that cell migration was increased *in vitro* when synovial MSCs were cultured in media supplemented with PDGF-BB. They also found that intra-articular PDGF-BB treatment increased MSC proliferation and colony-forming potential (53). Although beyond the scope of this study, increased levels of PDGF-BB in PL containing medias could provide an advantage when injecting MSCs intra-articularly by not only improving the function of injected MSCs but also recruitment and proliferation of endogenous MSCs already present within the joint. Previous studies have also shown the importance of TGF-β signaling in MSC differentiation, wherein the presence or overexpression of TGF-β improves chondrogenic differentiation of MSCs (54–56). Additionally, TGF-β can have an immunosuppressive effect, as it plays a critical role in dendritic cell tolerance and promoting regulatory dendritic cell generation (57–59). Not only has IL-10 been shown to upregulate Treg cells, it is a potent anti-inflammatory cytokine that plays a role in the pathogenesis of osteoarthritis (60). Future studies are needed to examine the role of these factors in platelet lysate on MSC differentiation capacity and immunomodulation *via* Treg cells.

We did not detect a distinct advantage of PPL over APL. While PPL contained the highest levels of IL-10, APL contained the highest concentrations of PDGF-BB and TGF-β. However, there were only significant differences when comparing these medias to FBS and ES. There were no significant differences between PPL and APL regarding T cell proliferation or cytokine production of BM-MSCs. Previous studies have shown that APL can have considerable variability depending on age, sex, and hydration status which may be counteracted by pooling PL from several individuals (11, 20). It is important to note that all horses in our study were young and healthy, therefore, some changes in growth factor and cytokine concentration may have been mitigated in APL than if older or systemically compromised horses were used.

A major limitation of the study is that the function of BM-MSCs cultured in different media types was only evaluated *in vitro* with T cell proliferation and analysis of cytokine production by BM-MSCs. Additional studies are required to further elucidate functional differences in BM-MSCs cultured in xenogen-free media *in vitro* and *in vivo*. Additional *in vitro* assays focused on function of these cells could include gene expression analysis and characterization of Treg transformation. Another limitation is that the quantification of proteins in the different medias should be interpreted with some caution as the immunoassays used were human or equine specific. Therefore, they could have different degrees of cross-reactivity with equine and bovine proteins skewing how much total protein was detected in each media.

## Conclusion

In conclusion, the results of this study showed that alternatives to FBS including APL, PPL and ES may be advantageous when culture expanding equine BM-MSCs. We found that PL supplemented medias had higher concentrations of PDGF, TGF-β, and IL-10, while BM-MSCs cultured in equine serum were the most effective at suppressing T cell proliferation. Additionally, IL-6 production was highest from BM-MSCs cultured in equine serum. Further studies are needed to further elucidate how each media affects BM-MSC function.

## Data availability statement

The raw data supporting the conclusions of this article will be made available by the authors, without undue reservation.

## Ethics statement

The animal study was reviewed and approved by IACUC University of Pennsylvania #806985.

## Author contributions

KE, RL, and KO contributed to study design, data collection, data analysis, and manuscript preparation. AG contributed to data collection, data analysis, and manuscript preparation. SC contributed to data collection. All authors approved the final manuscript.

## Funding

This study was funded by the Raymond Firestone Trust and Rakers/Tulleners Fund, University of Pennsylvania.

## Conflict of interest

The authors declare that the research was conducted in the absence of any commercial or financial relationships that could be construed as a potential conflict of interest.

## Publisher's note

All claims expressed in this article are solely those of the authors and do not necessarily represent those of their affiliated organizations, or those of the publisher, the editors and the reviewers. Any product that may be evaluated in this article, or claim that may be made by its manufacturer, is not guaranteed or endorsed by the publisher.
